# Comparison of Nutritional Flavor Substances in Meat Between Shanghai Local Pig Breeds and Commercial DLY Breed

**DOI:** 10.3390/foods14010063

**Published:** 2024-12-29

**Authors:** Yan Shi, Weilong Tu, Mengqian Cao, Lingwei Sun, Shushan Zhang, Jiehuan Xu, Mengqian He, Caifeng Wu, Defu Zhang, Jianjun Dai, Xinli Zhou, Jun Gao

**Affiliations:** 1 School of Health Science and Engineering, University of Shanghai for Science and Technology, Shanghai 200093, China; 233442683@st.usst.edu.cn; 2 Institute of Animal Husbandry and Veterinary Science, Shanghai Academy of Agricultural Sciences, Shanghai 201106, China; tuweilong@saas.sh.cn (W.T.); 13887223118@163.com (M.C.); sunlingwei1987@126.com (L.S.); smalltreexj@126.com (S.Z.); jiehuanxu810@163.com (J.X.); he1037247863@163.com (M.H.); wucaifengwcf@163.com (C.W.); zhangdefuzdf@163.com (D.Z.); daijianjun@saas.sh.cn (J.D.); 3 Key Laboratory of Livestock and Poultry Resources (Pig) Evaluation and Utilization, Ministry of Agriculture and Rural Affairs, Shanghai 201106, China; 4 Shanghai Municipal Key Laboratory of Agri-Genetics and Breeding, Shanghai 201106, China; 5 Shanghai Engineering Research Center of Breeding Pig, Shanghai 201106, China; 6 College of Fisheries and Life Science, Shanghai Ocean University, Shanghai 201306, China

**Keywords:** meat traits, nutritional flavor substances, amino acids, fatty acids, thiamine, inosine monophosphate

## Abstract

Chinese local pig breeds have unique meat flavor. In this study, we investigated the meat quality traits and the characteristics of the nutritional flavor substances such as amino acids (AAs), fatty acids (FAs), thiamine (Vitamin B1, VB1), and inosine monophosphate (IMP) in four Shanghai local pig breeds (MMS, SW, PD, and SHW) and the commercial crossbred Duroc × Landrace × Yorkshire (DLY) breed. The results showed that the intramuscular fat (IMF) and protein content in the longissimus dorsi muscle (L) of Shanghai local breeds, especially Shanghai MMS and PD breeds, were significantly higher than those of the DLY breed (*p*-value < 0.01). The inter-breed differences in amino acid (AA) content were even more significant in the gluteal muscle (G). Total amino acids (TAAs), flavor amino acids (FAAs), and essential amino acids (EAAs) were significantly higher in the G muscle of the four Shanghai local breeds than that in the DLY breed. The results for fatty acids (FAs) revealed that the differences in polyunsaturated fatty acids (PUFAs) were all highly significant (*p*-value < 0.0001), especially for the MMS breed, where the content of PUFAs in the L and G muscle amounted to (14.86 ± 2.06) g/100 g and (14.64 ± 2.83) g/100 g, respectively, which were significantly higher than those of other breeds. The MMS breed was also found to have the highest IMP content and the lowest thiamine (VB1) content among several pig breeds. Therefore, these differences in meat nutritional flavor substances provide new insights into the characterization of meat flavor in Shanghai local pig breeds.

## 1. Introduction

Pork is a commonly consumed red meat worldwide, and pork plays a key role in the human diet as an important source of high-biological-value proteins, essential fatty acids, vitamins (B group), and micronutrients such as zinc, selenium, and iron [1]. According to the Organization for Economic Cooperation and Development (OECD), China is the world’s largest producer and consumer of pork, with an annual per capita consumption of more than 30 kg [2]. Nutritional value and flavor characteristics are among the most important indicators of pork quality and influence consumer purchasing decisions [3,4]. Pork is an important part of people’s daily diet and has become one of the most consumed meats in the world, with its quality and nutritional value receiving widespread attention [5]. In the current context of increasing food safety and health awareness, people are paying more and more attention to the quality and nutritional value of food [6]. Currently, the largest market share is for ternary crossbred pigs (DLY), at nearly 90%. However, there are reports of poor meat quality and the presence of PSE (pale, soft, exudative) and DFD (dark, firm, dry) meat, which is prone to consumer dissatisfaction [7,8]. Chinese native pig breeds have unique characteristics, such as higher intramuscular fat (IMF) and better meat quality, which may be caused by long-term artificial or natural selection [8,9]. As one of China’s major markets and breeding centers, the Shanghai region has been highly prized for its relatively abundant local pig breeds with unique genetic backgrounds and growth characteristics. These include three of Shanghai’s indigenous pig breeds: Middle Meishan (MMS), Shawutou (SW), and Pudong White (PD), as well as a crossbred Shanghai White (SHW) from the 1970s [10]. However, to the best of our knowledge, there are no systematic studies and reports on the differences in meat nutrients and flavor substances between local Shanghai pig breeds and the DLY breed.

Pork contains many valuable nutrients such as proteins, lipids, fatty acid (FA), minerals, and vitamins [6]. The proteins and their component amino acids (AAs) found in pork can provide the body with essential amino acids (EAAs) [11]. Protein hydrolysis is one of the enzymatic reactions to derive flavor substances, which is influenced by endogenous proteases. AAs produced by protein hydrolysis also play an important role in pork flavor and nutrition, providing key nutritional values and helping to improve meat quality and flavor [12]. Some AAs, including Glu, Lys, Val, Ala and Leu, have been identified as important contributors to flavor [13] and become one of the most important indicators for evaluating the quality of meat. Peptides and free AAs have a taste-promoting effect in the maturation process of pork [14], Glu, Asp and glutamine help to accentuate the meat flavor and freshness characteristics of meat [15], Ile, Leu, and Phe have a bitter taste [16], and Ala, Ser, Thr, Gly, Pro, and hydroxyproline have a sweet taste [12].

Previous studies have shown that FAs, as important precursors, play a key role in the flavor profile of pork [14,17]. IMF is also one of the most important factors affecting the flavor and nutrition of pork meat, and low levels of IMF in cooked cured hams have been found to be associated with a reduction in volatile compounds and a pleasing aroma produced by the Maillard reaction [18]. In addition, the degree of lipid unsaturation is critical in determining the alteration of pork flavor caused by lipid oxidation, which plays a key role in aroma formation during storage and processing, and lipid-derived compounds, which are the main components of species-specific flavors [14,19,20]. And oxidation of a small portion of FAs can significantly alter the flavor of pork [5]. FA levels and composition are influenced by breed, species, fresh weight, post-slaughter senescence, and their interactions [21,22]. The lipid deposition and composition of pork not only affects the organoleptic quality, but also determines the nutritional quality of pork. There are three types of FAs in triglycerides, which are the main lipids in skeletal muscle fat, including saturated fatty acids (SFAs), monounsaturated fatty acids (MUFAs), and polyunsaturated fatty acids (PUFAs). PUFAs have been reported to have several health benefits [23].

Thiamine (vitamin B1, VB1) and its degradation products play a key role in the formation of the characteristic flavor of pork. Thermal decomposition and hydrolysis reactions of VB1 produce compounds that contribute significantly to the flavor characteristics of pork. VB1 can be degraded to produce meat flavor compounds and is an important source of typical flavor substances of roast pork, including furanone and furanethiol [24]. Inosine monophosphate (IMP) and its degradation products, ribose and hypoxanthine, are both considered important components in the formation and development of meat flavor, and the degradation of IMP to hypoxanthine may affect pork flavor [25]. IMP is one of the main contributors to pork flavor; it is a key quality characteristic that affects consumer preference. Interesting studies have analyzed the IMP content, screened out the important substances that distinguish Chinese pork from hybrid pork, and identified different pork muscles, and found that there are significant differences in IMP composition between breeds and muscles, which provides a theoretical basis for understanding the composition of flavor precursor substances and the identification of different muscles [26].

In this study, four local Shanghai pig breeds, MMS, SW, SHW, and PD, were used in this study, and we used the DLY as a control, which has a completely different flavor from the local pigs [27]. We analyzed the meat quality and nutritional flavor characteristics of Shanghai local pigs through the statistics of their meat quality traits and the determination of AAs, FAs, VB1, IMP, and other nutritional flavor substances in their muscles, to lay the foundation for meat quality evaluation and promote the local pig breeds in Shanghai.

## 2. Materials and Methods

### 2.1. Pork Samples

Pork samples from MMS, SW, SHW, and PD were obtained from designated local pig breeding farms in Shanghai, and DLY meat samples were obtained from Wufeng Shangshai Food Co.(Shanghai, China). The main ingredients of feed formulations for the pigs include crude protein 18.0%, leucine 0.63%, energy/MJ·kg 13.9%, methionine 0.29%, calcium 0.65%, threonine 0.56%, total phosphorus 0.49%, glutamic acid 2.91%, lysine 0.85%, and crude fiber 4%. All slaughtering was performed by licensed commercial slaughtering companies when the pigs reached the standardized market slaughter weight (100 ± 5 kg). Six pigs of each breed were each sampled, half male and half female, and the pork tissue collected from two parts, the longissimus dorsi muscle (L) and gluteal muscle (G). Muscle samples were taken from the left half of the carcass within 40 min after slaughter. The L muscle was sampled by stripping the sebaceous layer of the sampling site, removing the fat outside the meat-like fascia, transecting the L muscle of the back, and stripping the L muscle along the anterior end of the penultimate third thoracic vertebrae backward until the required length and weight of the sample was obtained. The G muscle sampling site was located at the top of the pig’s hind leg, just below the tip of the buttocks. The samples were put into plastic sealing bags and brought back to the laboratory under dry ice conditions.

### 2.2. Major Reagents and Instrumentation

Major reagents: 17 AA standards (purity ≥ 99.9%, wako, Japan), FA standards (Sigma-Aldrich, St. Louis, MO, USA), thiamine (VB1) standards (stock solution, purity ≥ 98%, Yuanyebio, Shanghai, China), 5-IMP (stock solution, purity ≥ 98%,Yuanyebio, Shanghai, China), HPLC grade acetonitrile (purity ≥ 99.9%, OCEANPAK company, Tianjin, China). HPLC system (Agilent Technologies series 1100, Wilmington, DE, USA), Sepax Amethyst C18-H (250 mm × 4.6 mm, 5 μm, Sepax Technologies, Inc., Newark, DE, USA), Xiang Yi High Speed Freezing Centrifuge (Changsha, Hunan, China).

### 2.3. Measurement of Meat Quality Traits

Meat quality traits were tested by the laboratory of Swine Genetic Breeding Research Laboratory, the Institute of Animal Husbandry and Veterinary Science, Shanghai Academy of Agricultural Sciences. Meat quality traits were determined with reference to the Chinese agricultural industry standard: Technical code of practice for pork quality assessment (Standard number: NY/T 821-2019). In this study, only meat quality traits were determined for L muscle. Measurement items included IMF, drip loss, pH_24h_, meat color, water content, electrical conductivity, hydrostatic pressure, fat content, protein content, and shear force. IMF content was measured by an automatic fat analyzer (XT10, ANKOM, Macedon, NY, USA) following the methods outlined in previous studies [28]. After thawing, the IMF content of 3 g of wet muscle tissue was quantified using petroleum ether extraction and the Soxhlet technique. Drip loss was calculated as the difference from the initial weight after 24 h. Briefly, cubic muscle samples of 4 cm in diameter were weighed 45 min after death and suspended in a plastic bag at 4 °C for 24 h. Subsequently, the muscle was removed from the bag and reweighed, and drip loss was determined by calculating the percentage change in weight. For pH determination according to the meat block method, the electrode was inserted directly into any measurement point at one end of the specimen for 30 s, and the displayed value was read and recorded.

Meat color was determined by a color difference analyzer (SP60, X-rite, Grand Rapids, MI, USA) after blooming of the meat to form oxymyoglobin at 4 °C. Electrical conductivity was determined using a carcass meat conductivity tester, calibrated before use. Fresh pork samples were weighed, and the conductivity electrode was inserted into the specimen for readings accurate to 0.01 mS/cm, with three parallel measurements on the same specimen. Hydrostatic pressure was determined using a manometer by weighing the mass of the specimen before pressurization, holding the pressure at 35 kg for 5 min, and then removing the pressure, removing the specimen and weighing it again; the hydrostatic pressure was calculated as the percentage change in weight.

The water content, fat content, and protein content of the pork cuts were analyzed using near-infrared reflectance transmission in a FoodScan device (S3000 NIR Technology Systems NSW, Australia). Each pork cut sample was ground according to AOAC Official Method 983.18, and approximately 100 g of the sample at 20 °C was placed in a glass circular cup for analysis. Shear force was measured using a muscle tenderness tester. Briefly, the muscle was heated in a water bath at 80 °C until the core temperature of the muscle reached 75 °C, then cooled at 4 °C for 24 h. The shear force of L muscle was averaged by five measurements using a tenderness meter (C-LM3B, Harbin, China).

### 2.4. Measurement of Amino Acids

Amino acids were determined according to high performance liquid chromatography. To a quantity of 1.0 g of sample was added 50 mL of 6 mol/L hydrochloric acid containing 0.1% phenol, and the sample was ground, then hydrolyzed at 110 °C for 24 h. After cooling, 1 mL of the hydrolyzed solution was taken and blown to near dryness with a nitrogen blower, dissolved with 1 ml of 0.1 M hydrochloric acid and filtered. A quantity of 200 μL of this solution and 200 μL of amino acid standard solution were combined in a 2 mL EP tube and 20 μL of n-leucine internal standard solution was added to each. Subsequently, 100 μL each of triethylamine acetonitrile and phenyl isothiocyanate acetonitrile solution were added to each tube, mixed, and left at 25 °C for 1 h. Then, 1 mL of n-hexane was added, shaken, and left for 10 min. The lower solution was diluted 5 times, filtered through a 0.22 μm filter, and tested on the machine.

### 2.5. Measurement of Fatty Acids

Fatty acids were determined with reference to the methods of previous studies [29]. To a quantity of 0.1 g~10 g homogeneous sample (weighed accurately to 0.1 mg) was added pyrogallic gallic acid and ethanol; this was mixed well and then hydrolyzed according to the specimen category. After hydrolysis, ethanol was added and transferred to a separatory funnel and rinsed several times with a mixture of ethyl ether and petroleum ether, and the fat was extracted. The extract was concentrated to dryness to obtain the fat extract. This was solubilized with n-hexane and treated with potassium hydroxide-methanol solution methyl esterification. After the reaction, water was added to the static stratification. This was centrifuged and the supernatant dilution was taken for gas chromatography–mass spectrometry determination.

### 2.6. Measurement of Inosine Monophosphate

IMP was determined with reference to previous studies [26] with minor modifications. Frozen pork samples were thawed in a refrigerated environment at 0 to 4 °C for 24 h. After thawing was completed, the meat samples were minced using a meat grinder and then 2.0 g of minced meat was accurately weighed (to an accuracy of 0.0001 g) into a 50-mL plastic centrifuge tube. Next, 10 mL of 6% perchloric acid solution was added to the tube for extraction. After thorough mixing in a vortex mixer, the tube was placed at 4 °C for 1 h and then centrifuged at 5000 rpm for 10 min. After centrifugation, the supernatant was removed, and the remaining solids were re-extracted, and the supernatants obtained from the two extractions were combined. The pH of the combined supernatant was adjusted to 6.5 using 3 moles per liter of sodium hydroxide solution and supplemented with water to reach a total volume of 50 mL, followed by thorough shaking. An appropriate amount of the supernatant was taken and filtered through a sterile hydrophilic microporous filter membrane of 0.22 μm pore size, and the filtrate was collected in a 2 mL sample vial and prepared for high performance liquid chromatography (HPLC) analysis. The samples were quantitatively analyzed by an external standard method. To qualitatively and quantitatively analyze IMP, inosine, and hypoxanthine in the samples, it was necessary to purchase the appropriate controls.

### 2.7. Measurement of Thiamine

Thiamine (VB1) was determined with reference to previous studies [30]. First, 5 g of the pork sample was mixed with 60 mL of hydrochloric acid at 0.1 molar concentration and hydrolyzed by heating at 121 °C for 30 min. After the hydrolysis was completed, the solution was allowed to cool naturally to room temperature, and then the pH of the solution was adjusted to 4.0 by adding a 2.0 molar concentration of sodium acetate solution Next, the enzyme mixture was added to the solution and incubated for 16 h at 37 °C. At the end of the incubation, the volume of the solution was fixed to 100 mL, followed by a filtration process. The filtered solution was extracted using potassium ferricyanide–sodium hydroxide solution. The extracted samples were analyzed by high-performance liquid chromatography (HPLC) on a chromatographic system equipped with a C18 column. The mobile phase for chromatographic analysis was a mixture of sodium acetate solution and methanol at a concentration of 0.05 molar, and the flow rate was set at 0.8 mL/min. The injection volume of the sample was 20 µL. In the fluorescence detector, the excitation wavelength was set at 375 nm and the emission wavelength was set at 435 nm. Finally, a standard curve for VB1 was established by determining the VB1 content in the sample for quantitative analysis.

### 2.8. Statistical Analysis

SPSS [31] Statistics for Windows, version 22.0, was utilized to perform the Student’s t-test for assessing the significance of differences among indicators. Statistical analysis of the data was carried out using GraphPad Prism [32] version 10.1.2 for Windows (GraphPad Software, San Diego, CA, USA). Comparisons between two groups were analyzed by *t*-test, and comparisons between multiple groups were analyzed by one-way ANOVA, and the results of the measurements were expressed as ”mean ± standard deviation”.

## 3. Results

### 3.1. Meat Quality Traits

The results of the tests of the meat quality traits of the five breeds are shown in Table 1. The IMFs of Shanghai local pig breeds were significantly higher (*p*-value < 0.01) than that of the DLY breed. In addition, in terms of meat color, the L* values of the four Shanghai local breeds of pigs were significantly higher than those of the DLY breed (*p*-value < 0.05); the a* values of the DLY pig breed were significantly higher than those of the Shanghai local breeds of pigs (*p*-value < 0.0001); the b* values of the Shanghai local pigs were significantly higher than those of the DLY breed, and the highest were found in the SHW breed (*p*-value < 0.01). Protein content was significantly higher in Shanghai native pigs than in DLY breed, and was highest in PD breed, followed by the SW, MMS, and SHW breeds (*p*-value < 0.0001).

### 3.2. Amino Acid

Seventeen AAs were detected in the muscles of all five breeds of pigs, DLY, MMS, SW, SHW, and PD, including seven EAAs, six major flavor amino acids (FAAs), and five other AAs, and the contents of various AAs are shown in Table 2 and Table 3.

In this study, it was found that Cys, Ile, and Val were significantly higher in the L muscle of the DLY breed than those of Shanghai local pig breeds (*p*-value < 0.01). Compared to the L muscle, this study found that in the G muscle, the total amino acids (TAAs), FAAs, and EAAs were significantly higher in the four Shanghai local breeds than in DLY, and most of the AAs detected were also significantly higher in Shanghai local breeds. For example, Asp and Glu, which are closely related to direct flavor presentation, were found to be (1.84 ± 0.10) and (2.83 ± 0.17) g/100 g in DLY, respectively, whereas among the four local breeds in Shanghai, the SHW had the highest Asp content of (2.15 ± 0.04) g/100 g, and the MMS the lowest (2.00 ± 0.07) g/100 g. Glu was the highest at (3.22 ± 0.07) g/100 g in SHW and the lowest at (3.08 ± 0.15) g/100 g in SW; these values were significantly higher than those of DLY in both groups. Sweetness-related AAs such as Ala, Ser, Thr, Gly, and Pro were also significantly higher than that of DLY.

### 3.3. Fatty Acid

Twenty-four fatty acids were detected in all five breeds of pigs, including 10 saturated fatty acids (SFAs) and 14 unsaturated fatty acids (UFAs), and the content of each FA is shown in Table 4 and Table 5. The resultant expression of FAs was calculated as the proportion of FA fractions to total FAs.

According to the FA results (Table 4 and Table 5), there was a similar trend in the muscle of all breeds of pigs. Of the FAs, oleic and palmitic acids are the highest, followed by linoleic and stearic acids. The data also showed that the differences in PUFAs were all highly significant (*p*-value < 0.01), with the MMS breed having the highest level, at (14.86 ± 2.06) g/100 g in L muscle and (14.64 ± 2.83) g/100 g in G muscle, respectively. The amount of linoleic acid differed significantly (*p*-value < 0.05) between breeds, with higher levels in the Shanghai indigenous pig breeds than in the DLY breed.

### 3.4. VB1 and IMP

**Figure 1:**
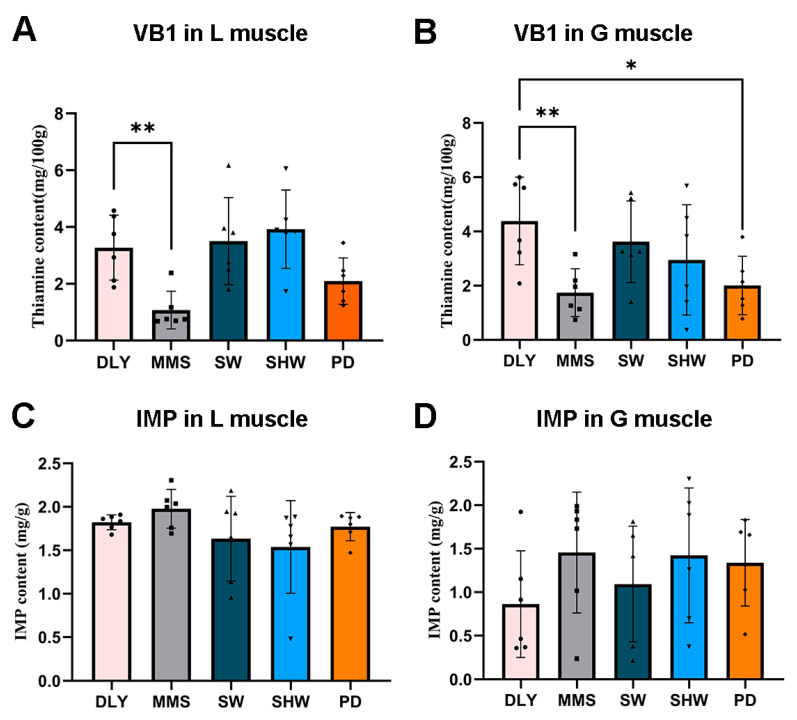
VB1 and IMP content in the L and G muscle among pig breeds. “*” indicates that the *p*-value < 0.05 and “**” indicates that the *p*-value < 0.01.

In this study, VB1 and IMP content in the L and G muscle of five pig breeds were determined (Figure 1). In the comparison between Shanghai local pig breeds and the DLY breed, it was found that in the L muscle (Figure 1A), VB1 content in the DLY breed was significantly higher than that in the MMS breed (*p*-value < 0.01). In the G muscle, VB1 content was also significantly higher (*p* < 0.05) in the DLY breed than that in the MMS and PD breeds (Figure 1B). In contrast to VB1, the highest levels of IMP in the muscle of the five pig breeds were found in the MMS breed, but did not reach significant difference (Figure 1C,D).

## 4. Discussion

### 4.1. Meat Quality Traits

Meat quality is important to consumers, as they prefer pork with higher quality of tenderness, juiciness, and flavor [33]. Indicators for evaluating pork quality include meat color, juiciness, IMF content, pH value, tenderness, flavor, etc. [34]. IMF is the total amount of fat located between the muscle fibers and the lipid droplets in the muscle cells [35]. IMF content is closely related to the eating quality of pork and can affect, among other things, the tenderness of pork [36]. IMF content and FA composition are the main factors influencing meat quality [37]. In addition, restrictions on the finishing stage of the meat would reduce the IMF content [38]. Local pig breeds are a great resource for improving meat quality because they have a greater ability to deposit fat into muscle [8,39]. For example, the Xiangcun Black Pig, which is the offspring of the Taoyuan Black Pig and the Duroc, is different from the Duroc, Yorkshire, and Long White Pigs in that they are usually well adapted to environmental conditions and more often show higher meat quality [40]. IMF is also an important organoleptic quality of pork that influences consumer preference [41].

China’s local pig breeds are rich in resources, and the IMF content of most of the local pigs is higher than that of the DLY commercial pigs. For example, the IMF content of the Beijing Black Stream and the Laiwu Black Pig averaged 4.2%, and the IMF content was significantly higher than that of the DLY pigs (*p*-value< 0.05) [42]. The IMF content of the Xiangcun black pig breed can reach up to 5.42%, and high IMF content in meat is believed to result in more flavor [43]. In this study, four local breeds of Shanghai pigs (MMS, SW, SHW, and PD) were analyzed for meat quality traits, and it was found that the IMF content of the four local breeds was significantly higher than that of the DLY commercial pigs. The results of the analyses showed that among the four local breeds, only the pH and water content were not significantly different; all other indexes were significantly different. The meat quality of MMS and PD is better than that of the other two breeds. SHW is a breed bred from Shanghai local pig breeds and Landrace pigs in the 1970s, so its meat quality is between those of Shanghai local pigs and foreign lean pigs, and the results also show that its IMF content is higher than that of DLY but lower than that of the other three Shanghai local pig breeds.

Meat color is one of the most important meat characteristics that affects the desire to eat it, and it has been suggested that myoglobin (Mb) is the main chemical component that determines meat color, and that the post-slaughter time is the most important factor affecting meat color [44,45]. In this study, we found that there were also some significant differences in meat color between Shanghai local pigs and DLY pig breeds. Protein is an important nutrient in people’s diet and is one of the most important parameters for assessing pork quality and nutrition [46]. In this study, the protein content of Shanghai local pork was significantly higher than that of DLY pig breeds, as was the case for IMF, which indicates that Shanghai local pig breeds have an advantage over lean commercial pig breeds in terms of meat quality traits. Drip loss is the percentage of weight of muscle lost under gravity during a certain period of time after death due to the release of water and protein during the aging process of meat [47]. Drip loss is an indicator used to predict water-holding capacity, as high drip loss is associated with low water-holding capacity [48]. Higher drip loss leads to poor meat quality and loss of nutrients in pork. In this study, the drip loss of PD and MMS from Shanghai local pigs was significantly lower than that of the other three pig breeds, indicating that PD and MMS have better water-holding capacity and meat quality.

### 4.2. Amino Acids

The TAAs and FAAs were generally higher in the G muscle of the four Shanghai local pig breeds than in DLY, whereas this difference was not significant in the L muscle. For example, in the G muscle, the Asp and Glu contents of Shanghai local pigs were significantly (*p*-value < 0.001) higher than those of DLY, while they were not significant (*p*-value > 0.05) in the L muscle. Among the many FAAs that have been studied and reported, Glu and Asp are notable for their close association with pork freshness, and they are recognized as the most representative FAAs that play a key role [49]. Researchers investigated the role of functional AAs such as Arg, Leu, and Glu in regulating adipose tissue development and lipid metabolism, and improving meat quality and nutritional value in pigs [50,51,52]. Leu is a functional amino acid with a carbon skeleton that is converted in muscle to acetyl coenzyme A and acetoacetate for FA synthesis [53]. In addition, Leu is used to improve pork quality and promote green and healthy production [54], The results showed that the addition of 0.14% Leu to the diet of fattening pigs promoted the deposition of IMF in pork [53]. Numerous studies have proven that the FAA content and TAA content of local pigs are higher than those of commercial pigs [26,55,56,57,58,59]. The present study revealed that the total FAAs in the G muscle of Shanghai local pig breeds was higher than that of DLY pig breeds, and the key AAs, such as Asp and Glu, showed consistent characteristics, suggesting that Shanghai local pig breeds have better flavor performance in terms of nutritive value and flavor of the G muscle.

### 4.3. Fatty Acid

Fatty acids play a key role in providing energy in the human body and can be subdivided into three categories based on the degree of saturation of their carbon–hydrogen bonds: SFAs, MUFAs, and PUFAs. The nutritional value and flavor of pork are strongly influenced by the type and content of these FAs [37]. The results of this study revealed that the meat samples of Shanghai local pig breeds, especially MMS, had higher PUFAs than DLY pig breeds. It is worth noting the PUFAs, which are straight-chain FAs characterized by the presence of two or more double bonds, possess a variety of health benefits, such as lowering cholesterol levels, regulating blood lipids, and lowering blood pressure, and anti-tumor, vision protection, anti-inflammatory, anti-oxidative and anti-inflammatory effects [60]. The physiological role of PUFAs in dietary composition has been extensively studied, suggesting that their metabolism may influence clinical conditions associated with inflammatory and oxidative processes. Experimental evidence suggests that PUFAs can enhance the expression and activity of antioxidant enzymes through various signaling pathways, including superoxide dismutase, catalase, and glutathione peroxidase [61].

A variety of PUFAs, including linoleic acid, α-linolenic acid, docosahexaenoic acid, and arachidonic acid, can be found in pork from Shanghai local pig breeds. Among them, linoleic acid is the main PUFA in pork and is one of the essential fatty acids which helps to soften blood vessels, prevent cardiovascular diseases, and provide important benefits for human health [62,63,64,65,66,67,68]. In addition, significant differences in the FA composition of different pork masses have been demonstrated in previous studies, and analyzing such significant differences can also help in the screening and identification of different pork muscles [26]. There are a number of influences that contribute to differences in nutritional flavor substances between breeds, especially differences in fatty acid composition, which may be influenced by the diet in addition to the genetic factors of the breed, and although the main nutrients in swine farm feed formulations are more consistent, it has not been possible to achieve complete control.

### 4.4. VB1 and IMP

VB1 is a vitamin essential for normal cellular function. It exists as the free form monophosphate, diphosphate, or triphosphate. VB1 plays a special role in the body as a coenzyme necessary for the metabolism of carbohydrates, fats, and proteins [69]. Thermal decomposition of VB1 releases aromas that add a distinctive flavor to meat, and VB1 accumulation may also be a reliable indicator of post-slaughter pork aging [70]. The structure and properties of VB1 varied among breeds and there were significant differences between breeds; some studies have proven that there are obvious differences in FAs, VB1, and IMP in meat samples between breeds, which provides a theoretical basis for the identification of muscles of different breeds [26,71]. Similar results were obtained in the present study, where the content of VB1 in the muscle of MMS and PD breeds was lower than those of other breeds; however, their IMP content was relatively higher.

Aroma has been shown to be an important factor in determining the acceptability of pork products and generally comes from a variety of volatile compounds [26]. Raw (or uncooked) pork is considered to be odorless, whereas it constitutes a source of rich constituents (e.g., lipids, AAs, reducing sugars, and VB1) called aroma precursors. During thermal cooking, the above precursors produce volatile compounds through lipid oxidation and degradation, the Merad reaction, interactions between lipid oxidation and Merad, and VB1 degradation [72]. Precursors may vary between pig breeds. Chinese black and Laiwu pigs were distinguished from DLY pigs by identifying their characteristic fatty acids and unique odorants [17]. It has also been demonstrated that the VB1 content of pork meat from different breeds of pigs is characterized differently, with lower reducing sugars and higher VB1 content being characteristic of Tibetan pork [30]. The VB1 content of pork varied between breeds in this study, and the VB1 content in L and G muscle differed significantly between breeds of pigs, producing potentially different aromas and possibly different precursors, and this significant difference could be one of the bases for distinguishing the meat quality of different breeds of pork.

IMP is an important fresh taste substance in animal tissues, which is closely related to the production of fresh taste in livestock and poultry meat foods, and it was only before the discovery of the fresh taste receptor that freshness was considered as a basic taste, mainly known as the taste of monosodium glutamate [73,74,75,76,77]. Since IMP is a major contributor to meat flavor, it is widely used as an important indicator for assessing meat flavor, and the function of IMP to increase food flavor is widely recognized [78]. Pork flavor is produced by many substances, either by themselves or by their interactions. FAs and IMP are two compounds that affect meat flavor. In addition, IMP is a major flavorful nucleotide substance and has become an important indicator of meat flavor [79]. Excessive amounts of inosine and hypoxanthine as metabolites of IMP lead to the production of a bitter flavor in meat [25].

Among the pig breeds compared in this study, IMP levels in MMS pork were relatively the highest, but did not reach significant levels compared to the DLY group. The reason for this is the relatively large variation between individuals within pig breeds, which may be constrained by the sample size. It has also been suggested that the amount of IMP in meat is affected by factors such as species, sex, age, muscle type, and pre-slaughter processing methods [80,81]. Some researchers have studied the breast and leg meat of local chickens and broilers and found that the IMP content of different muscles of different breeds of chickens is different [82]. It has also been suggested that IMP is one of the main contributors to pork flavor, and it can be one of the indicators for identifying different breeds of pork [26].

## 5. Conclusions

In this study, the meat quality traits, as well as nutritional flavor substances, of DLY commercial pigs and four local pig breeds in Shanghai were comprehensively measured and analyzed, revealing the significant advantages of Shanghai local pigs in terms of meat quality and flavor. The experimental results showed that the muscles of local Shanghai pigs contain high levels of AAs and FAs, which significantly enhance the nutritional value and flavor characteristics of their meat. The meat of Shanghai local pigs is rich in flavorful AAs, monounsaturated fatty acids, VB1, and IMP, which are important sources of flavor for their meat. Meanwhile, the higher IMF and protein contents of Shanghai local pig breeds indicated that Shanghai local pigs had better meat quality performance. The present study revealed the characteristics of meat nutritional flavor substances of different pig breeds, especially Shanghai local pig breeds, in terms of differences in breed genetic backgrounds and identified some candidate flavor biomarkers. To some extent, the study provides some referable data for the future selection of pig breeds, especially the meat quality of local pig breeds in Shanghai.

## Figures and Tables

**Table 1:** Results of measurement of meat quality traits for L muscle.

Traits	DLY	MMS	SW	SHW	PD	*p*-Value
IMF (%)	3.10 ± 0.82	5.55 ± 0.53	5.11 ± 1.13	4.03 ± 2.53	5.82 ± 0.74	**
Drip loss (%)	5.62 ± 0.94	3.55 ± 1.18	5.83 ± 0.17	5.57 ± 1.52	3.78 ± 1.21	**
pH_24h_	5.84 ± 0.38	5.71 ± 0.06	5.49 ± 0.28	5.70 ± 0.32	5.44 ± 0.11	ns
Lightness (L*)	41.80 ± 3.69	44.66 ± 1.74	49.91 ± 5.21	47.57 ± 7.75	42.57 ± 3.75	*
Redness (a*)	10.67 ± 1.58	3.46 ± 3.04	9.03 ± 3.09	5.77 ± 3.35	2.87 ± 1.70	****
Yellowness (b*)	6.28 ± 1.93	8.91 ± 1.49	9.86 ± 2.86	10.45 ± 1.13	8.67 ± 1.20	**
WC (%)	68.78 ± 1.72	68.57 ± 3.01	66.70 ± 7.43	67.93 ± 1.13	68.17 ± 1.96	ns
EC (ms/cm)	1.31 ± 0.01	3.40 ± 1.65	1.25 ± 0.29	2.25 ± 2.54	3.40 ± 1.29	*
YP (%)	84.50 ± 5.18	94.61 ± 3.14	88.01 ± 6.55	90.10 ± 5.67	91.34 ± 2.56	*
FC (%)	19.62 ± 0.37	13.56 ± 2.32	14.70 ± 6.32	19.47 ± 0.26	12.30 ± 2.43	***
PC (%)	11.53 ± 2.10	17.89 ± 3.81	18.60 ± 2.46	12.58 ± 1.40	19.55 ± 0.46	****
Shear force (N)	3.66 ± 0.66	2.45 ± 0.32	4.28 ± 2.07	3.69 ± 0.53	2.52 ± 0.54	*

Note: IMF: intramuscular fat, WC: water content, EC: electrical conductivity, YP: hydrostatic pressure, FC: fat content, and PC: protein content. “ns” indicates that the *p*-value > 0.05. “*” indicates that the *p*-value < 0.05 and “**” indicates that the *p*-value < 0.01. “***” and “****” indicate highly significant differences, with *p*-value values less than 0.001 and 0.0001, respectively. The values are presented by means ± standard deviations.

**Table 2:** Amino acid content of the L muscle of the meat.

Amino Acid	DLY	MMS	SW	SHW	PD	*p*-Value
Asp	1.95 ± 0.09	1.87 ± 0.04	1.92 ± 0.06	1.89 ± 0.04	1.91 ± 0.05	ns
Glu	3.01 ± 0.13	2.90 ± 0.04	2.99 ± 0.10	2.90 ± 0.07	2.92 ± 0.06	ns
Ser	0.70 ± 0.04	0.68 ± 0.02	0.69 ± 0.02	0.68 ± 0.02	0.69 ± 0.02	ns
Gly	0.68 ± 0.02	0.66 ± 0.03	0.67 ± 0.02	0.68 ± 0.05	0.69 ± 0.03	ns
His	0.76 ± 0.05	0.76 ± 0.03	0.63 ± 0.12	0.74 ± 0.07	0.76 ± 0.03	*
Arg	1.19 ± 0.06	1.13 ± 0.02	1.16 ± 0.04	1.14 ± 0.03	1.14 ± 0.03	ns
Thr	0.77 ± 0.05	0.74 ± 0.01	0.74 ± 0.03	0.72 ± 0.02	0.72 ± 0.02	*
Ala	0.99 ± 0.05	0.95 ± 0.02	0.96 ± 0.03	0.95 ± 0.03	0.95 ± 0.03	ns
Pro	0.58 ± 0.02	0.56 ± 0.02	0.58 ± 0.01	0.60 ± 0.03	0.61 ± 0.02	*
Tyr	0.54 ± 0.04	0.51 ± 0.01	0.52 ± 0.02	0.51 ± 0.02	0.51 ± 0.02	ns
Val	0.77 ± 0.05	0.70 ± 0.02	0.72 ± 0.03	0.69 ± 0.03	0.68 ± 0.03	**
Met	0.43 ± 0.04	0.38 ± 0.03	0.40 ± 0.03	0.40 ± 0.03	0.37 ± 0.02	*
Cys	0.09 ± 0.01	0.06 ± 0.01	0.05 ± 0.01	0.04 ± 0.01	0.02 ± 0.01	****
Ile	0.74 ± 0.05	0.67 ± 0.02	0.68 ± 0.03	0.65 ± 0.03	0.63 ± 0.02	****
Leu	1.26 ± 0.09	1.24 ± 0.03	1.24 ± 0.05	1.23 ± 0.05	1.22 ± 0.04	ns
Phe	0.60 ± 0.04	0.57 ± 0.01	0.58 ± 0.03	0.56 ± 0.02	0.56 ± 0.02	ns
Lys	1.63 ± 0.10	1.54 ± 0.03	1.57 ± 0.07	1.53 ± 0.06	1.52 ± 0.05	*
TAA	16.73 ± 0.91	15.91 ± 0.30	16.09 ± 0.65	15.92 ± 0.50	15.90 ± 0.44	ns
EAA	6.25 ± 0.42	5.83 ± 0.12	5.93 ± 0.27	5.80 ± 0.22	5.70 ± 0.19	*
FAA	7.77 ± 0.36	7.45 ± 0.14	7.63 ± 0.25	7.49 ± 0.18	7.54 ± 0.18	ns
EAA/TAA (%)	37.34 ± 0.50	36.66 ± 0.25	36.85 ± 0.28	36.42 ± 0.50	35.82 ± 0.33	****
FAA/TAA (%)	46.47 ± 0.38	46.84 ± 0.23	47.46 ± 0.49	47.09 ± 0.47	47.45 ± 0.23	***

Notes: Asp: Aspartic acid, Glu: Glutamic acid, Ser: Serine, Gly: Glycine, His: Histidine, Arg: Arginine, Thr: Threonine, Ala: Alanine, Pro: Proline, Tyr: Tyrosine, Val: Valine; Met: Methionine, Cys: Cystine, Ile: Isoleucine; Leu: leucine, Phe: Phenylalanine, Lys: Lysine, TAA**:** total amino acids, EAA**:** essential amino acids, and FAA: flavor amino acids. “ns” indicates that the *p*-value > 0.05. “*” indicates that the *p*-value < 0.05 and “**” indicates that the *p*-value < 0.01. “***” and “****” indicate highly significant differences, with *p*-value values less than 0.001 and 0.0001, respectively. The values are presented by means ± standard deviations.

**Table 3:** Amino acid content of the G muscle of the meat.

Amino Acid	DLY	MMS	SW	SHW	PD	*p*-Value
Asp	1.84 ± 0.10	2.00 ± 0.07	2.02 ± 0.07	2.15 ± 0.04	2.02 ± 0.07	***
Glu	2.83 ± 0.17	3.12 ± 0.09	3.08 ± 0.15	3.22 ± 0.07	3.11 ± 0.09	***
Ser	0.65 ± 0.04	0.69 ± 0.03	0.70 ± 0.03	0.76 ± 0.01	0.72 ± 0.03	****
Gly	0.71 ± 0.04	0.74 ± 0.07	0.75 ± 0.05	0.82 ± 0.04	0.78 ± 0.05	*
His	0.66 ± 0.06	0.67 ± 0.13	0.62 ± 0.13	0.84 ± 0.08	0.69 ± 0.13	*
Arg	1.12 ± 0.07	1.21 ± 0.05	1.20 ± 0.06	1.28 ± 0.02	1.21 ± 0.04	***
Thr	0.68 ± 0.05	0.74 ± 0.04	0.73 ± 0.04	0.79 ± 0.01	0.73 ± 0.04	**
Ala	0.92 ± 0.05	1.00 ± 0.04	0.99 ± 0.05	0.60 ± 0.03	1.01 ± 0.04	****
Pro	0.62 ± 0.05	0.66 ± 0.05	0.65 ± 0.04	0.71 ± 0.03	0.71 ± 0.03	**
Tyr	0.48 ± 0.03	0.52 ± 0.03	0.52 ± 0.03	0.56 ± 0.02	0.53 ± 0.03	**
Val	0.68 ± 0.04	0.76 ± 0.04	0.74 ± 0.07	0.74 ± 0.05	0.66 ± 0.08	*
Met	0.34 ± 0.04	0.35 ± 0.04	0.37 ± 0.02	0.43 ± 0.02	0.38 ± 0.03	***
Cys	0.01 ± 0.01	0.03 ± 0.01	0.02 ± 0.01	0.02 ± 0.02	0.01 ± 0.05	ns
Ile	0.64 ± 0.05	0.71 ± 0.05	0.67 ± 0.05	0.68 ± 0.04	0.61 ± 0.07	*
Leu	1.17 ± 0.08	1.28 ± 0.07	1.26 ± 0.08	1.34 ± 0.04	1.27 ± 0.06	**
Phe	0.54 ± 0.04	0.60 ± 0.03	0.59 ± 0.04	0.63 ± 0.02	0.59 ± 0.03	**
Lys	1.45 ± 0.10	1.62 ± 0.09	1.59 ± 0.09	1.70 ± 0.04	1.59 ± 0.08	***
TAA	15.35 ± 0.97	16.69 ± 0.76	16.52 ± 0.86	17.74 ± 0.31	16.61 ± 0.76	***
EAA	5.50 ± 0.40	6.05 ± 0.36	5.95 ± 0.35	6.30 ± 0.16	5.82 ± 0.36	**
FAA	7.32 ± 0.42	7.98 ± 0.27	7.96 ± 0.37	8.45 ± 0.16	8.03 ± 0.26	****
EAA/TAA (%)	35.85 ± 0.41	36.22 ± 0.67	36.07 ± 0.04	35.53 ± 0.47	35.03 ± 0.82	*
FAA/TAA (%)	47.71 ± 0.40	47.84 ± 0.97	48.22 ± 0.64	47.67 ± 0.70	48.40 ± 0.69	ns

Notes: Asp: Aspartic acid, Glu: Glutamic acid, Ser: Serine, Gly: Glycine, His: Histidine, Arg: Arginine, Thr: Threonine, Ala: Alanine, Pro: Proline, Tyr: Tyrosine, Val: Valine; Met: Methionine, Cys: Cystine, Ile: Isoleucine; Leu: leucine, Phe: Phenylalanine, Lys: Lysine, TAA: total amino acids, EAA: essential amino acids, and FAA: flavor amino acids. “ns” indicates that the *p*-value > 0.05. “*” indicates that the *p*-value < 0.05 and “**” indicates that the *p*-value < 0.01. “***” and “****” indicate highly significant differences, with *p*-value values less than 0.001 and 0.0001, respectively. The values are presented by means ± standard deviations

**Table 4:** Fatty acid content of the L muscle (%FA).

Sort of Fatty Acid	DLY	MMS	SW	SHW	PD	*p*-Value
Capric acid	0.17 ± 0.09	0.10 ± 0.01	0.07 ± 0.02	0.10 ± 0.01	0.11 ± 0.01	ns
Lauric acid	0.09 ± 0.03	0.09 ± 0.01	0.08 ± 0.02	0.08 ± 0.01	0.09 ± 0.01	ns
Myristic acid	1.38 ± 0.11	1.64 ± 0.26	1.36 ± 0.25	1.22 ± 0.15	1.53 ± 0.15	ns
Myristoleic acid	0.04 ± 0.02	0.02 ± 0.01	0.02 ± 0.01	0.01 ± 0.01	0.03 ± 0.01	*
Pentadecanoic acid	0.08 ± 0.04	0.06 ± 0.01	0.03 ± 0.01	0.02 ± 0.01	0.03 ± 0.01	ns
Palmitic acid	29.89 ± 1.64	28.59 ± 1.30	28.39 ± 1.50	26.93 ± 1.32	29.70 ± 1.20	ns
Cis-7-Hexadecenoic acid	0.56 ± 0.45	0.29 ± 0.07	0.22 ± 0.02	0.25 ± 0.06	0.33 ± 0.07	ns
Palmitoleic acid	3.19 ± 0.43	2.58 ± 0.55	2.28 ± 0.56	1.82 ± 0.53	3.11 ± 0.51	ns
14-Methylhexadecanoic acid	0.05 ± 0.02	0.02 ± 0.01	0.03 ± 0.01	0.02 ± 0.02	0.04 ± 0.01	ns
Margaric acid	0.19 ± 0.03	0.30 ± 0.08	0.15 ± 0.03	0.21 ± 0.07	0.25 ± 0.05	ns
Cis-10-Heptadecenoic acid	0.14 ± 0.03	0.23 ± 0.06	0.10 ± 0.02	0.16 ± 0.06	0.23 ± 0.06	ns
Stearic acid	15.19 ± 3.13	22.15 ± 6.10	28.84 ± 3.10	31.55 ± 10.34	25.58 ± 3.98	ns
Oleic acid	34.89 ± 1.45	25.18 ± 5.15	24.70 ± 3.43	25.52 ± 7.22	25.81 ± 6.22	ns
Elaidic acid	3.09 ± 0.46	2.07 ± 0.70	1.76 ± 0.35	1.59 ± 0.87	2.45 ± 0.57	ns
Cis-11-Octadecenoic acid	0.09 ± 0.02	0.09 ± 0.02	0.07 ± 0.02	0.06 ± 0.02	0.11 ± 0.02	ns
Linoleic acid	6.94 ± 1.29	13.13 ± 1.88	8.78 ± 1.63	7.25 ± 2.39	7.49 ± 1.19	*
Linolenic acid	0.50 ± 0.33	0.70 ± 0.09	0.59 ± 0.09	0.40 ± 0.22	0.40 ± 0.07	*
Nonadecanoic acid	0.87 ± 0.57	0.30 ± 0.11	0.21 ± 0.04	0.28 ± 0.15	0.30 ± 0.18	ns
Cis-10-Nonadecenoic acid	0.10 ± 0.02	0.10 ± 0.04	0.08 ± 0.01	0.10 ± 0.03	0.10 ± 0.03	ns
Arachidic acid	0.41 ± 0.10	0.37 ± 0.08	0.47 ± 0.05	0.40 ± 0.08	0.49 ± 0.11	ns
Cis-11-Eicosenoic acid	1.05 ± 0.56	0.96 ± 0.12	1.00 ± 0.26	1.22 ± 0.24	1.18 ± 0.11	ns
11,14-Eicosadienoic acid	0.46 ± 0.60	0.57 ± 0.14	0.45 ± 0.10	0.44 ± 0.09	0.39 ± 0.07	ns
8,11,14-Eicosatrienoic acid	0.14 ± 0.03	0.14 ± 0.03	0.10 ± 0.01	0.10 ± 0.03	0.11 ± 0.03	ns
Arachidonic acid	0.66 ± 0.41	0.33 ± 0.06	0.19 ± 0.08	0.27 ± 0.09	0.26 ± 0.07	ns
SFA	48.24 ± 1.44	53.62 ± 5.30	59.66 ± 2.21	60.81 ± 8.84	58.12 ± 7.20	**
UFA	51.76 ± 1.44	46.38 ± 5.30	40.34 ± 2.21	39.19 ± 8.84	41.88 ± 7.20	**
MUFA	49.30 ± 1.97	31.52 ± 5.81	30.22 ± 3.69	30.72 ± 8.28	33.23 ± 6.85	**
PUFA	8.68 ± 1.41	14.86 ± 2.06	10.12 ± 1.76	8.47 ± 2.72	8.65 ± 1.31	****

Notes: SFA: saturated fatty acid, UFA: unsaturated fatty acid, MUFA: monounsaturated fatty acids, and PUFA: polyunsaturated fatty acids. “ns” indicates that the *p*-value > 0.05. “*” indicates that the *p*-value < 0.05 and “**” indicates that the *p*-value < 0.01. “****” indicate highly significant differences, with *p*-value values less than 0.001 and 0.0001, respectively. The values are presented by means ± standard deviations.

**Table 5:** Fatty acid content in G muscle (%FA).

Sort of Fatty Acid	DLY	MMS	SW	SHW	PD	*p*-Value
Capric acid	0.02 ± 0.03	0.16 ± 0.02	0.14 ± 0.03	0.11 ± 0.02	0.13 ± 0.03	**
Lauric acid	0.14 ± 0.02	0.14 ± 0.02	0.12 ± 0.02	0.08 ± 0.01	0.10 ± 0.02	*
Myristic acid	1.50 ± 0.09	1.68 ± 0.15	1.40 ± 0.26	1.18 ± 0.08	1.76 ± 0.49	**
Myristoleic acid	0.03 ± 0.01	0.25 ± 0.53	0.02 ± 0.00	0.02 ± 0.00	0.03 ± 0.01	ns
Pentadecanoic acid	0.15 ± 0.08	0.08 ± 0.09	0.06 ± 0.07	0.06 ± 0.09	0.10 ± 0.12	**
Palmitic acid	27.49 ± 1.06	28.50 ± 1.03	27.85 ± 1.45	25.64 ± 0.60	30.08 ± 3.55	**
Cis-7-Hexadecenoic acid	0.70 ± 0.18	0.63 ± 0.23	0.50 ± 0.14	0.32 ± 0.14	0.29 ± 0.16	*
Palmitoleic acid	3.44 ± 0.47	3.03 ± 0.56	2.94 ± 0.55	2.13 ± 0.44	3.58 ± 0.51	**
14-Methylhexadecanoic acid	0.10 ± 0.03	0.06 ± 0.03	0.08 ± 0.02	0.02 ± 0.01	0.04 ± 0.01	***
Margaric acid	0.23 ± 0.03	0.35 ± 0.10	0.19 ± 0.01	0.24 ± 0.07	0.32 ± 0.10	**
Cis-10-Heptadecenoic acid	0.19 ± 0.06	0.34 ± 0.13	0.15 ± 0.02	0.17 ± 0.06	0.28 ± 0.11	**
Stearic acid	14.51 ± 1.89	13.53 ± 2.61	15.36 ± 3.33	30.54 ± 9.55	21.21 ± 8.76	*
Oleic acid	34.88 ± 1.05	29.90 ± 0.76	32.84 ± 3.41	27.25 ± 8.59	26.93 ± 6.71	****
Elaidic acid	3.43 ± 0.42	3.04 ± 0.36	2.99 ± 0.42	2.10 ± 0.55	2.91 ± 1.03	***
Cis-11-Octadecenoic acid	0.11 ± 0.01	0.10 ± 0.03	0.09 ± 0.01	0.08 ± 0.02	0.14 ± 0.03	**
Linoleic acid	7.28 ± 0.84	12.6 ± 12.50	10.05 ± 1.37	6.99 ± 0.89	8.27 ± 1.34	*
Linolenic acid	0.76 ± 0.53	0.83 ± 0.18	0.79 ± 0.05	0.37 ± 0.09	0.51 ± 0.10	ns
Nonadecanoic acid	1.53 ± 0.64	1.26 ± 0.68	1.35 ± 0.56	0.35 ± 0.25	0.59 ± 0.47	ns
Cis-10-Nonadecenoic acid	0.38 ± 0.17	0.25 ± 0.09	0.00 ± 0.00	0.09 ± 0.04	0.11 ± 0.06	*
Arachidic acid	1.10 ± 0.41	0.94 ± 0.49	0.85 ± 0.27	0.41 ± 0.14	0.53 ± 0.25	**
Cis-11-Eicosenoic acid	1.14 ± 0.22	1.21 ± 0.29	1.16 ± 0.25	1.17 ± 0.07	1.16 ± 0.12	**
11,14-Eicosadienoic acid	0.19 ± 0.07	0.37 ± 0.15	0.33 ± 0.14	0.41 ± 0.10	0.39 ± 0.15	*
8,11,14-Eicosatrienoic acid	0.25 ± 0.07	0.28 ± 0.10	0.23 ± 0.10	0.12 ± 0.06	0.16 ± 0.08	*
Arachidonic acid	0.89 ± 0.25	0.82 ± 0.34	0.65 ± 0.18	0.36 ± 0.18	0.44 ± 0.23	**
SFA	46.69 ± 1.97	46.66 ± 2.78	47.38 ± 3.28	58.63 ± 8.95	54.84 ± 7.65	**
UFA	53.31 ± 1.97	53.34 ± 2.78	52.62 ± 3.28	41.37 ± 8.95	45.16 ± 7.65	**
MUFA	44.06 ± 1.75	38.71 ± 1.75	40.68 ± 4.13	33.18 ± 9.32	35.38 ± 7.97	*
PUFA	9.26 ± 1.24	14.64 ± 2.83	11.94 ± 1.36	8.19 ± 1.18	9.78 ± 1.33	****

Notes: SFA: saturated fatty acid, UFA: unsaturated fatty acid, MUFA: monounsaturated fatty acids, and PUFA: polyunsaturated fatty acids. “ns” indicates that the *p*-value > 0.05. “*” indicates that the *p*-value < 0.05 and “**” indicates that the *p*-value < 0.01. “***” and “****” indicate highly significant differences, with *p*-value values less than 0.001 and 0.0001, respectively. The values are presented by means ± standard deviations.

## Data Availability

The original contributions presented in the study are included in the article, further inquiries can be directed to the corresponding authors.
